# HISSTA: a human in situ single-cell transcriptome atlas

**DOI:** 10.1093/bioinformatics/btaf142

**Published:** 2025-03-31

**Authors:** Jiwon Yu, Jiwoo Moon, Minseo Kim, Gyeol Han, Insu Jang, Jinyoung Lim, Seungmook Lee, Seok-Hwan Yoon, Woong-Yang Park, Byungwook Lee, Sanghyuk Lee

**Affiliations:** Department of Life Science, Ewha Womans University, Seoul 03760, Republic of Korea; Department of Life Science, Ewha Womans University, Seoul 03760, Republic of Korea; Korean Bioinformation Center (KOBIC), Korean Research Institute of Bioscience and Biotechnology, Daejeon 34141, Republic of Korea; Department of Life Science, Ewha Womans University, Seoul 03760, Republic of Korea; Korean Bioinformation Center (KOBIC), Korean Research Institute of Bioscience and Biotechnology, Daejeon 34141, Republic of Korea; R&D Division, Geninus Inc., Seoul 05836, Republic of Korea; R&D Division, Geninus Inc., Seoul 05836, Republic of Korea; R&D Division, Geninus Inc., Seoul 05836, Republic of Korea; R&D Division, Geninus Inc., Seoul 05836, Republic of Korea; GxD Inc., Kashiwa, Chiba 277-0882, Japan; Korean Bioinformation Center (KOBIC), Korean Research Institute of Bioscience and Biotechnology, Daejeon 34141, Republic of Korea; Department of Life Science, Ewha Womans University, Seoul 03760, Republic of Korea

## Abstract

**Motivation:**

Spatial transcriptomics holds great promise for revolutionizing biology and medicine by providing gene expression profiles with spatial information. Until recently, spatial resolution has been limited, but advances in high-throughput in situ imaging technologies now offer new opportunities by covering thousands of genes at a single-cell or even subcellular resolution, necessitating databases dedicated to comprehensive coverage and analysis with user-friendly intefaces.

**Results:**

We introduce the HISSTA database, which facilitates the archival and analysis of in situ transcriptome data at single-cell resolution from various human tissues. We have collected and annotated spatial transcriptome data generated by MERFISH, CosMx SMI, and Xenium techniques, encompassing 112 samples and 28 million cells across 16 tissue types from 63 studies. To decipher spatial contexts, we have implemented advanced tools for cell type annotation, spatial colocalization, spatial cellular communication, and niche analyses. Notably, all datasets and annotations are interactively accessible through Vitessce, allowing users to focus on regions of interest and examine gene expression in detail. HISSTA is a unique database designed to manage the rapidly growing dataset of in situ transcriptomes at single-cell resolution. Given its comprehensive data content and advanced analysis tools with interactive visualizations, HISSTA is poised to significantly impact cancer diagnosis, precision medicine, and digital pathology.

**Availability and implementation:**

HISSTA is freely accessible at https://kbds.re.kr/hissta/. The source code is available at https://doi.org/10.5281/zenodo.14904523.

## 1 Introduction

Recent advancements in spatial transcriptome (ST) technologies are revolutionizing research in biology and medicine. These technologies enable the analysis of gene expression with spatial precision, enhancing our understanding of biological processes and medical conditions where positional information is crucial. Mainstream ST platforms primarily utilize two approaches: deep RNA sequencing of specific spots and high-throughput in situ imaging using high-resolution microscopy.

The first approach provides transcriptome-wide expression profiles at varying spatial resolutions. For example, the Visium platform from 10x Genomics offers transcriptome profiling at 50 μm resolution (Ståhl *et al.* 2016) and, more recently, 2 μm resolution with its HD service. Chen *et al.* achieved an impressive 0.22 μm resolution with Stereo-seq, allowing a subcellular view of mouse organogenesis (Chen *et al.* 2022). However, these array-based ST technologies often struggle to accurately deduce expression profiles at the single-cell level due to difficulties in determining cell boundaries and differences between cell sizes and spot sizes.

In contrast, in situ transcriptome technologies, relying on microscope imaging, enable direct transfer of spatial information to the cellular or even subcellular level, provided boundary information is available. This boundary information can be experimentally determined or computationally inferred. MERFISH, developed by Chen *et al.* (2015) and commercialized by Vizgen, was a pioneering effort in this category, followed by CosMx SMI from Nanostring (Merritt *et al.* 2020) and Xenium from 10x Genomics (Salas *et al.* 2025). Earlier versions supported a few hundred genes, but gene contents are rapidly expanding. For instance, Nanostring has demonstrated a whole transcriptome panel with over 18 000 RNA targets for pancreatic cancer tissue using CosMx SMI, while 10x Genomics has launched the Xenium Prime 5K service, covering approximately 5000 genes.

Several databases exist for spatial omics data, varying in modality, gene content, resolution, and sample types. Databases like SpatialDB (Fan *et al.* 2020), SPASCER (Fan *et al.* 2023), STOmicsDB (Xu *et al.* 2024), SORC (Zhou *et al.* 2024), and SCAR (Deng *et al.* 2024) contain transcriptome sequencing data based on low-resolution arrays and thus do not truly offer single-cell resolution spatial data. CROST (Wang *et al.* 2024) and SOAR (Li *et al.* 2022) include both array-based deep sequencing data and in situ spatial data for humans, but the portion of in situ methods is relatively minor, and advanced functional analyses, such as cell–cell interaction mapping or colocalization, are not supported. The absence of ST databases dedicated to in situ imaging-based methods is due to these technologies being recently commercialized. The rapid technological advancements and intense competition are leading to an exponential increase in data volume, highlighting the urgent need for archival databases and analytical methods to support the growing research demands.

In response, we have developed HISSTA, a comprehensive database focused on human ST data at single-cell resolution using in situ imaging methods. HISSTA encompasses the largest volume of such datasets and supports a series of advanced tools for the analysis and visualization of spatial contexts in the samples.

## 2 Materials and methods

### 2.1 Data collection—spatial transcriptome

We collected spatial transcriptome datasets generated using in situ imaging methods, specifically, MERFISH (Vizgen), CosMx SMI (Nanostring), and Xenium (10x Genomics). Public archival sites [NCBI GEO (Edgar *et al.* 2002), Zenodo (https://zenodo.org/), DBKERO (Suzuki *et al.* 2018), Dryad (White *et al.* 2008), and bioRxiv (Sever *et al.* 2019)] were searched using keywords such as “human,” “MERFISH,” “Xenium,” “CosMx,” “spatial transcriptomics,” “spatial transcriptome,” and “in situ.” Search results were manually reviewed to confirm relevance and data availability.

### 2.2 Data processing—dimensionality reduction and clustering

Each spatial transcriptome dataset was analyzed using the Seurat R package (v5.0.1) (Butler *et al.* 2018). Cells with fewer than 20 transcripts and 10 genes were excluded based on quality control criteria. The raw count matrix was normalized using the “SCTransform” function. Dimensionality reduction was performed through principal component analysis (PCA), with the number of principal components selected automatically by a custom routine. Cells were then clustered using the Louvain algorithm at four different resolutions (0.3, 0.5, 0.7, and 1.0) to accommodate various user needs. Finally, the UMAP algorithm was used for cluster visualization.

### 2.3 Reference scRNA-seq compendium

For annotating cell types in spatial transcriptome data, HISSTA requires marker genes and reference expression profiles for each cell type.

We constructed a compendium of reference scRNA-seq datasets by collecting representative datasets for each cell type from the Human Cell Atlas (HCA) (Regev *et al.* 2017), CellTypist (Xu *et al.* 2023), and relevant publications (Supplementary Table S1). All datasets are from healthy human samples, except for ovary tissue, where only a tumor dataset was available. To leverage domain expertise and mitigate tissue-dependent complexities, we included datasets with available cell type annotations. Several source databases were already integrations of public datasets from multiple cohorts. Our reference single-cell compendium included 4 076 981 cells from 531 human donors across 16 tissue types.

### 2.4 Hierarchical classification of cell types

Due to the limited gene coverage in most public in situ ST data, high-resolution cell type assignments can be challenging. We developed a hierarchical classification system incorporating public annotation results from the reference scRNA-seq compendium. This system includes 4 or 5 levels, depending on tissue types, with primary levels categorized into epithelial, stromal, and immune cell types. The secondary level, which is tissue-specific, is the main focus for cell type annotation in in situ ST data. For example, in breast tissue, epithelial cells are divided into luminal and myoepithelial cells; immune cells into B cells, plasma cells, pDCs, DCs, mast cells, macrophages, monocytes, neutrophils, and TNKILC cells; and stromal cells into endothelial cells, fibroblasts, pericytes, and VSMCs. Classification details are provided in Supplementary File S1.

### 2.5 Cell type annotation of reference scRNA-seq compendium

Cell type annotation results from source databases were critically evaluated for integration into HISSTA. For the secondary level annotation of ST datasets, cell types from classification levels 3–5 were merged into the secondary level. Cells with uncertain or unreported types, as well as those with inconsistent hierarchical annotations, were removed. Additionally, rare cell types at the secondary level were filtered out, and ambiguous hierarchical classifications were discarded to maintain clarity in our cell type hierarchy.

### 2.6 Marker genes for cell type annotation of in situ ST data

HISSTA maintains a list of cell type marker genes for annotating *in situ* ST data. These markers were identified at the secondary cell type level from reference scRNA-seq data using Seurat’s “FindAllMarkers” function, which compares a cell group of interest (cg) with all other cells (og). Genes not present in the gene panel were excluded, so marker genes are specific to gene panel of *in situ* ST data. We then evaluated the percent difference in gene expression between the two groups (pct.diff(pct.cg – pct.og) > 0.2) to ensure enrichment in the cell cluster. Candidate markers were ranked by average fold change, and up to 20 genes with an average log2FC > 1 were selected. Canonical cell type markers from the original database or literature were added to the list after critical review. For tumor tissue samples, we also added “malignant” markers encompassing well-known cancer-associated markers such as KRT and CEACAM, as well as tissue-specific cancer markers from literature. The list of canonical and malignant marker genes is available on the website.

### 2.7 Spatial context analyses

Based on the cell type annotation results, HISSTA offers diverse methods of analyzing cellular contexts, including spatial colocalization, spatial cellular communication, and spatial niche analyses. First, spatial colocalization of different cell types was calculated using hoodscanR (v1.0.0) (Liu *et al.* 2024). Spatially neighboring cells were identified using the “findNearCells” function to locate 500 neighboring cells, and colocalization correlations between different cell types was calculated.

Next, we used CellChat (v2.1.2) (Jin *et al.* 2025) to investigate spatially proximal cell–cell communications. All cell–cell communication pathways in the CellChat database were used to count the number of interactions and interaction weights between different cell types. Outgoing or incoming signals were separately evaluated to help decipher intricate cellular communication networks.

Cell niche analysis identifies regions characterized by specific combinations of various spatially adjacent cell types, providing cellular context with respect to spatial microenvironments. We used the “BuildNicheAssay” function of Seurat v5 with the number of niches and neighbors set to 5 and 30, respectively. Pathway activity scores were calculated for each identified cell niche using the ssGSEA algorithm with the hallmark gene sets from the MSigDB database (v7.5.1) (Liberzon *et al.* 2015).

### 2.8 Interactive visualization with Vitessce

The Seurat object containing analysis results was converted into an Anndata object (Wolf *et al.* 2018) and stored in Zarr format. When available, cell segmentation information from the original dataset was saved in JSON format. If such information was not available, we generated a diamond-shaped boundary centered on the cell coordinate, which was stored within the AnnData object.

### 2.9 Database and web development

The HISSTA system is designed with a three-layer architecture comprising the data, logic, and presentation layers. The data layer acts as the core database, storing and managing experimental ST datasets, various analysis results, and reference scRNA-seq datasets. It also includes a file-based ST database to be used for Vitessce (Keller *et al.* 2025) visualization. Oracle MySQL (v5.7.40) is used for database management, while the Linux file system supports the Vitessce service. This structure enables efficient storage and management of large-scale data, along with rapid search and retrieval.

The logic layer serves as a middleware, facilitating communication between the data and presentation layers. It is responsible for implementing data processing and business logic. We used the Spring Framework based on Java 1.8 for main development, and the MyBatis framework for database interaction. MyBatis provides high-performance mapping between object-oriented programming and relational databases, efficiently handling complex data requests. The logic layer is designed to manage multiple concurrent data requests, ensuring system scalability while maintaining accuracy and efficiency in data processing.

The presentation layer provides the interface for user interaction and supports various visualization functionalities offered by HISSTA. The web interface is developed using jQuery.js (v3.5), Bootstrap (v5.3.3), and HTML5, while interactive visualization is implemented through the Vitessce JavaScript API (v3.4.6). The Vitessce API enhances visualization performance for large-scale data by utilizing the AnnData-Zarr format.

The HISSTA web system has been tested across multiple web browsers, including Safari, Chrome, and Edge, on Mac, Windows, and Linux environments.

## 3 Database content

### 3.1 Overview

**Figure 1.**
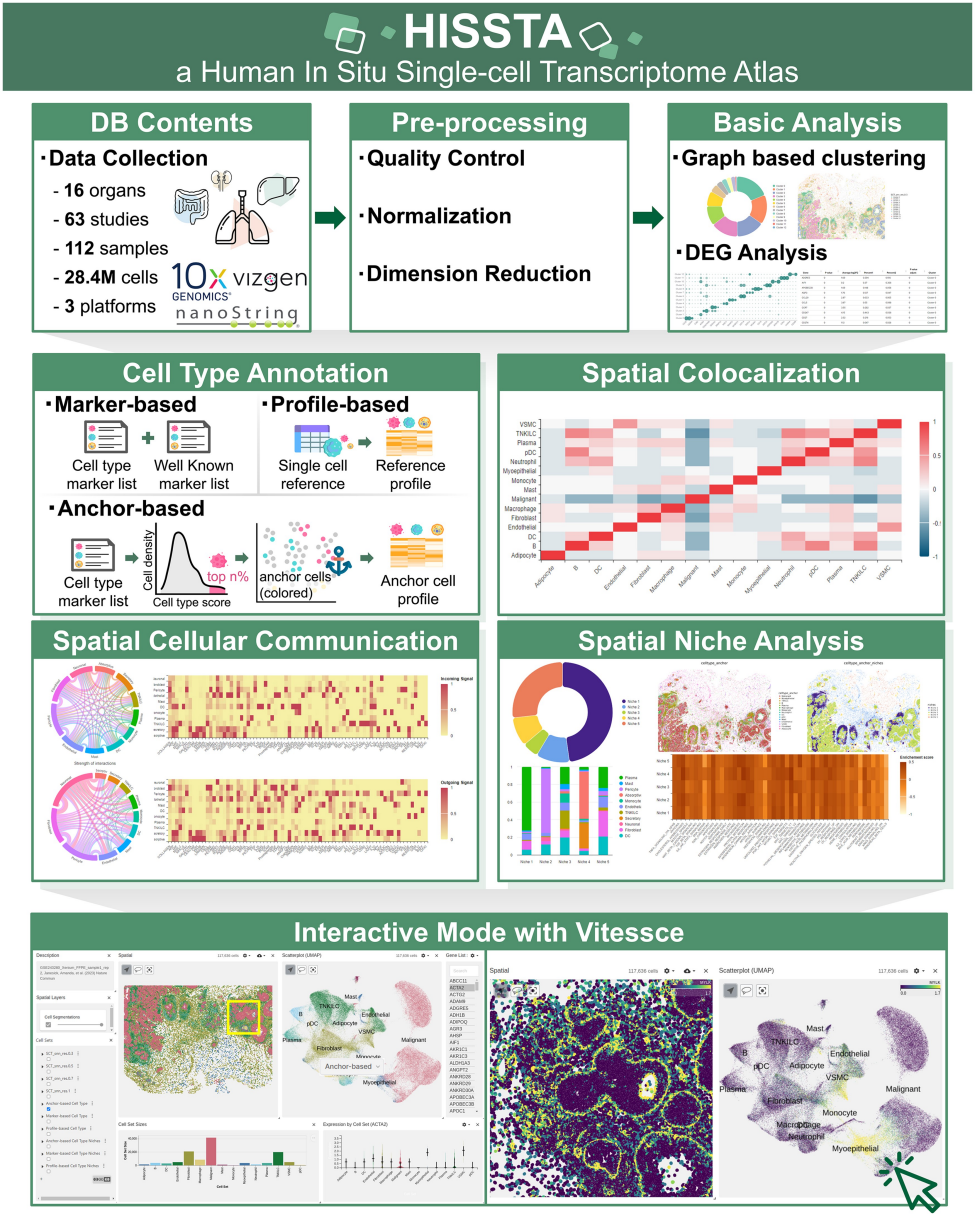
Overview of HISSTA.

HISSTA offers a user-friendly platform for the in-depth analysis and exploration of in situ spatial transcriptome (ST) data at single-cell resolution (Fig. 1). We have collected in situ transcriptome data generated using advanced labeling and imaging technologies like MERFISH, CosMx SMI, and Xenium. The database comprises 112 samples and over 28.4 million cells across 16 diverse human tissues collected from 63 studies. These datasets were subjected to basic analyses such as quality control, normalization, dimensionality reduction, graph-based clustering, and differential gene expression analysis. We made extensive efforts for robust and reliable annotation of cell types by supporting a novel anchor-based method as well as marker-based and profile-based methods. Furthermore, we have implemented advanced analyses such as spatial colocalization, spatial cellular communication, and spatial niche analyses. Given the importance of visual exploration of ST data, we implemented Vitessce, which allows users to select specific clusters or genes and visually examine their spatial expression patterns.

### 3.2 Cell type annotation

**Figure 2.**
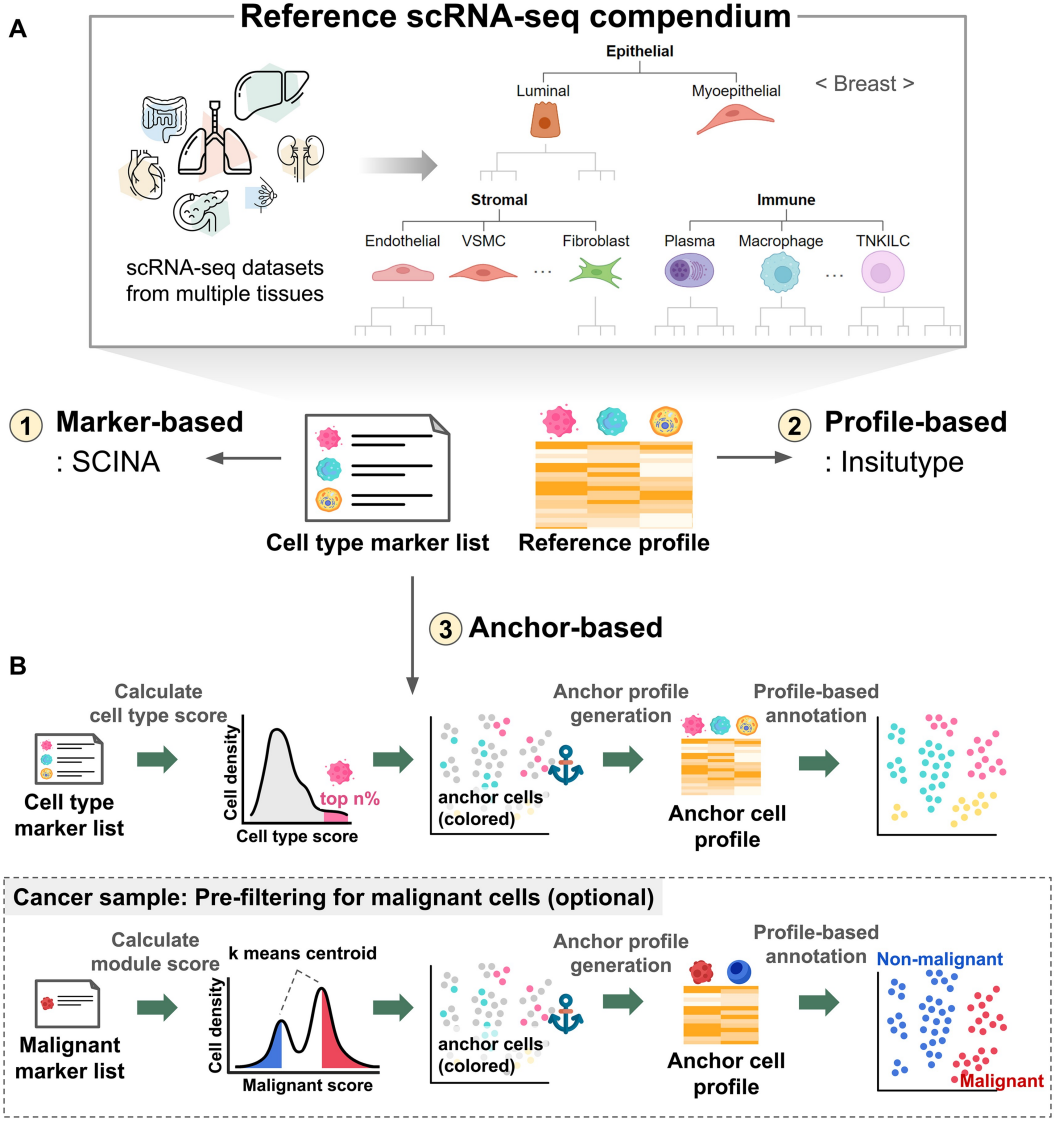
Reference scRNA-seq compendium and cell type annotation. (A) Construction of a compendium of reference scRNA-seq datasets. Cell type hierarchy at the primary and secondary levels is shown for breast tissue. (B) Cell type annotation methods in HISSTA.

Cell type annotation is fundamental to single-cell data analysis. Accurate assignment becomes more challenging for in situ ST data due to the limited number of gene probes, which are only a few hundred in most public datasets. To meet the diverse needs of users, HISSTA supports three methods of cell type annotation: marker-based, profile-based, and anchor-based (Fig. 2).

All these methods require marker gene lists and/or expression profiles as references. We built a compendium of scRNA-seq reference datasets that provide reliable results for cell type annotation, including HCA for various tissues, CellTypist, and relevant publications (Supplementary Table S1). Additionally, we established a hierarchical classification system of cell types with 4 or 5 levels, considering the annotation results (Fig. 2A). The primary level included epithelial cells, stromal cells, and immune cells, while the secondary level included further details depending on the tissue types (Supplementary File S1). Our annotation target was focused on the secondary level, where stromal cells were divided into fibroblasts, endothelial cells, and several types of vascular cells, and immune cells into macrophages, neutrophils, dendritic cells, B cells, etc. Details on producing marker gene lists and reference profiles are described in the Methods section.

Marker-based cell type annotation is performed using the SCINA tool (v1.2.0) (Zhang *et al.* 2019), a semi-supervised model utilizing prior knowledge of signature genes through an expectation-maximization algorithm. Among the panel genes of the dataset, up to 20 marker genes were selected from the tissue-matching reference scRNA-seq dataset for each cell type and fed into the SCINA tool, with the default options except convergence_rate = 0.999, sensitivity_cutoff = 0.9, allow_unknown = 0, and rm_overlap = 0.

For profile-based annotation, we used Insitutype (v1.0.0) (Danaher *et al.* 2022), a likelihood-based model specifically designed for cell type annotation of single-cell ST data. Reference expression profiles were created by calculating the average expression values for each cell type at the secondary level from the tissue-matching reference scRNA-seq data.

The anchor-based method is a novel hybrid algorithm combining the simplicity of the marker-based method and the reliability of the profile-based method. The main idea, inspired by Shiau’s work (Shiau *et al.* 2024), is to first identify “anchor cells,” which are defined as cells whose cell type can be designated unambiguously, and use these reliable anchor cells for cell type annotation of the remaining cells using a profile-based method (Fig. 2B). Specifically, cell type scores were calculated from marker gene expression, and only top 20% of cells were designated as anchor cells. Then, a gene expression profile was produced from the average expression of anchor cells for each cell type. Resulting profiles were subsequently used in the profile-based annotation of the remaining cells using Insitutype. For tumor samples, we adopted a pre-filtering step to identify malignant cells, where marker gene expression of malignant genes was used for binary classification of malignant and normal cells. Here, k-means (k = 2) clustering was used to identify two centroids, and two outlier cell groups were defined as anchor cells for normal and malignant cells. Insitutype was again used to identify all malignant cells. Other cells were regarded as normal cells and were subject to the main anchor-based cell type annotation.

## 4 User interface

### 4.1 Data browse and statistics

**Figure 3.**
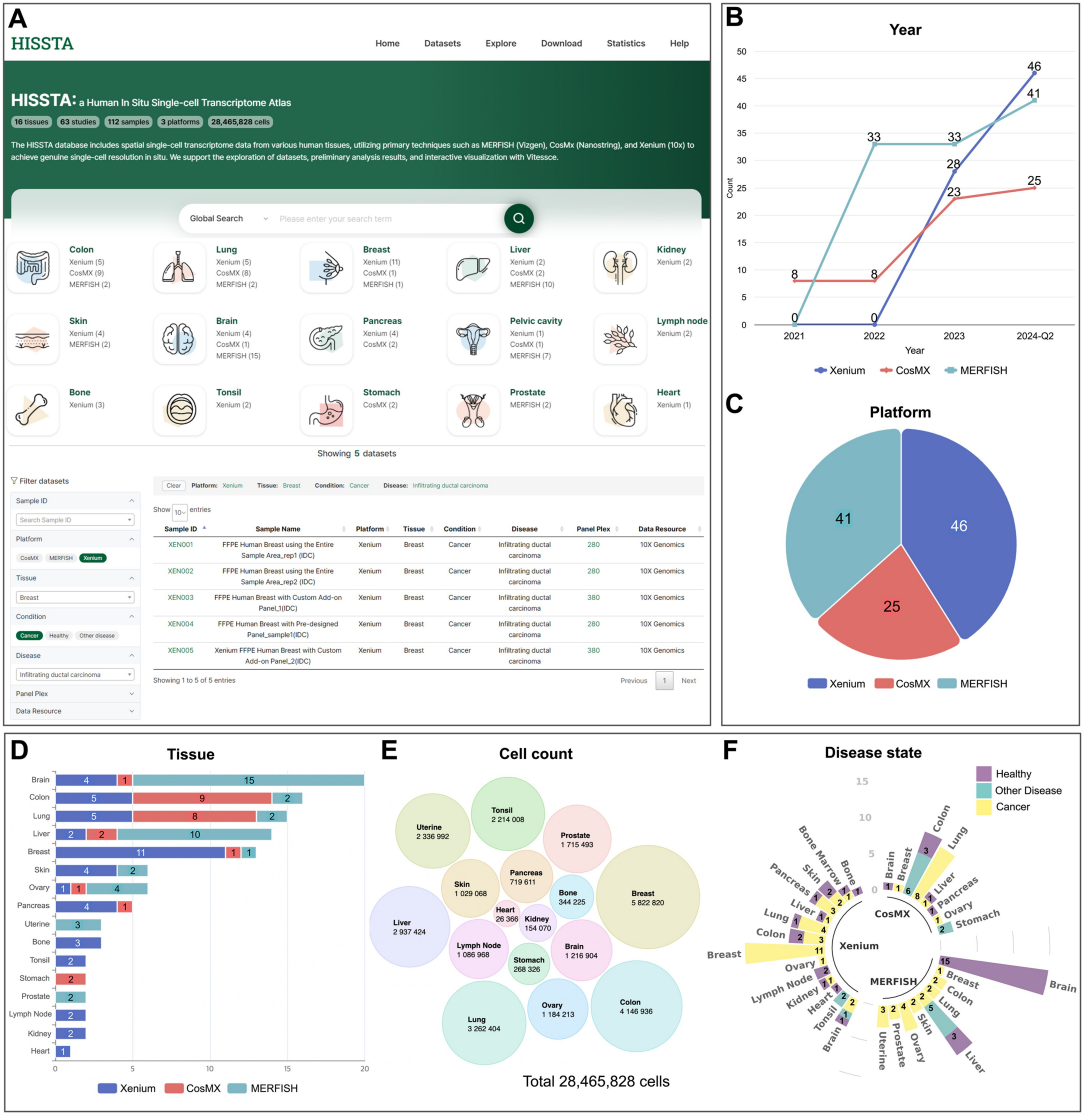
Screenshots from HISSTA search and statistics pages. (A) Homepage and datasets. Note that the filter and search functions are available on the Datasets page. (B) Data amount by year and platform. The count numbers are cumulative. (C) Ratio of datasets across different platforms. (D) Number of datasets across 16 tissues. Colors indicate different platforms. (E) Cell counts across 16 tissues. (F) Dataset coverage among disease states.

HISSTA supports various methods of searching and browsing database contents. All datasets are curated based on sample characteristics such as tissue and disease, as well as experimental conditions such as platform and panel plex. These curated datasets can be accessed via the organ icons showing the number of available datasets on different platforms (Fig. 3A). The dataset menu displays all datasets with detailed information listed in table format. Filter and search functions are available to quickly select datasets of interest. Clicking the sample ID shows detailed information and analysis results for that specific sample, as described in the following section. Clicking the gene plex number allows users to download and examine the gene list of the panel.

The statistics page shows various statistics of HISSTA datasets. The amount of ST data is rapidly increasing for all three platforms, as shown in the yearly statistics, with Xenium data leading recent growth (Fig. 3B and C). Statistics by tissue type reveal that the available data are most abundant for brain tissue, followed by colon, lung, liver, and breast (Fig. 3D). However, cell counts are largest for breast tissue, followed by colon, lung, liver, and others (Fig. 3E). Looking into disease states, breast and lung datasets are mostly cancer tissues, whereas brain datasets are primarily from health normal tissues (Fig. 3F).

### 4.2 Dataset information and analysis results

**Figure 4.**
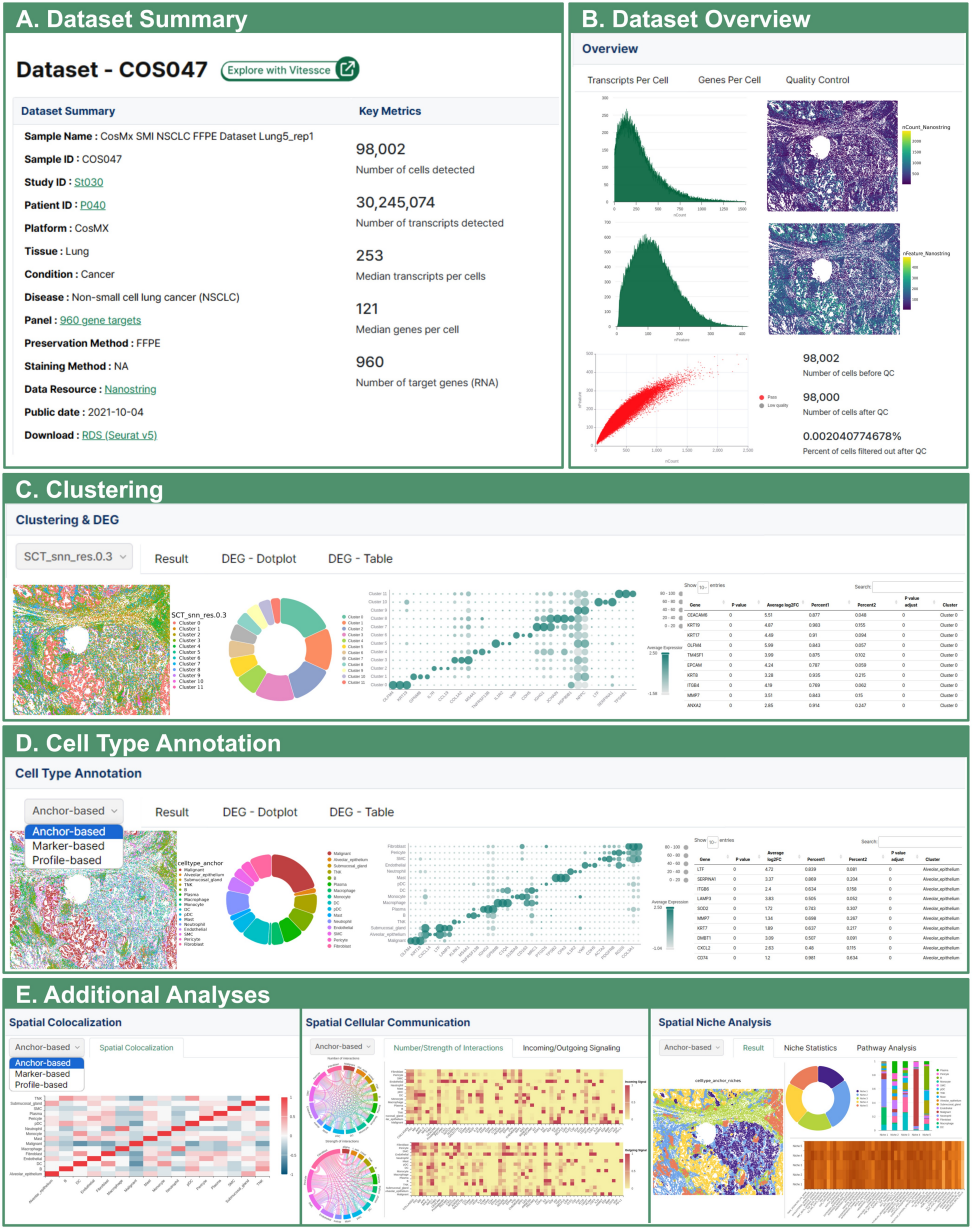
Screenshots from HISSTA results pages. (A) Dataset Summary for a lung cancer sample, generated using CosMx SMI technology. Characteristics of the sample and experimental methods (e.g. 960-plex CosMx panel) are shown, along with key metrics after data pre-processing. Note that HISSTA provides a link to Vitessce for interactive visual analysis and a link for downloading the Seurat object in RDS format for further analysis. (B) Dataset overview information for quality control assessment. (C) Clustering and DEG analysis results. (D) Cell type annotation results. These results are available as a colored tissue image map, a pie chart of cell type ratios, a dot plot of representative marker genes, and a table to examine signature genes for each cell cluster. (E) Results from advanced spatial context analyses. Most images can be downloaded.

All datasets in HISSTA are analyzed with our pipeline consisting of quality control, normalization, dimensionality reduction and clustering, cell type annotation, and advanced spatial context analyses. Analysis results are available in a user-friendly GUI. The dataset summary provides detailed information such as sample name, disease, panel name, and preservation method. It also includes key metrics such as the number of detected cells, the number of detected transcripts, the median transcripts per cell, and the median genes per cell (Fig. 4A). In the dataset overview, users can examine the distribution of transcripts per cell and genes per cell in the raw data, as well as identify low-quality cells that were removed based on quality control criteria (Fig. 4B).

Understanding spatial patterns and differences in gene expression within cells or tissues is essential for the analysis of spatial data. In the clustering menu, results at four different resolutions (0.3, 0.5, 0.7, and 1.0) are visualized using spatial image plots and pie charts, with differential gene expression (DEG) plots and tables (Fig. 4C). Accurate identification of cell types is the most fundamental and crucial step. Results from three methods of cell type annotation are visually presented in a similar manner (Fig. 4D). These spatial visualizations are critical for understanding the spatial contexts in terms of cell clustering and cell type annotation results.

HISSTA supports additional advanced analyses of spatial contexts, including spatial colocalization, spatial cellular communications, and cell niches (Fig. 4E). Spatial colocalization analysis by hoodscanR identifies neighborhood cells and calculate colocalization correlations between different cell types. CellChat v2 is used to identify spatially proximal cell–cell communications. Interaction numbers and strengths between different cell types are visualized with circular and heatmap plots. Incoming and outgoing signals are also presented in a separate tab. Unlike these methods that treat each cell independently, cell niche analysis by “BuildNicheAssay” function of Seurat identifies anatomical regions composed of dynamically interacting entities, thus better describing microenvironments. Resulting niches are further analyzed for specific enrichment of cell type composition or biological pathways. Pathway enrichment scores from the ssGSEA method are visualized as a heatmap. These spatial context analyses are particularly useful for understanding detailed cellular interactions and their roles in spatial contexts such as tumor microenvironments.

### 4.3 Interactive visualization and exploration with Vitessce

Visual exploration of specific regions is a crucial capability for analyzing spatial images of biological data. Vitessce is a visual integration tool designed for exploring spatial single-cell experiments, developed by the Gehlenborg lab and used in the HuBMAP Portal (Jain *et al.* 2023). It responds quickly enough to be embedded in web documents and supports basic visualization functionalities such as zooming, panning, and multi-window interactions.

**Figure 5.**
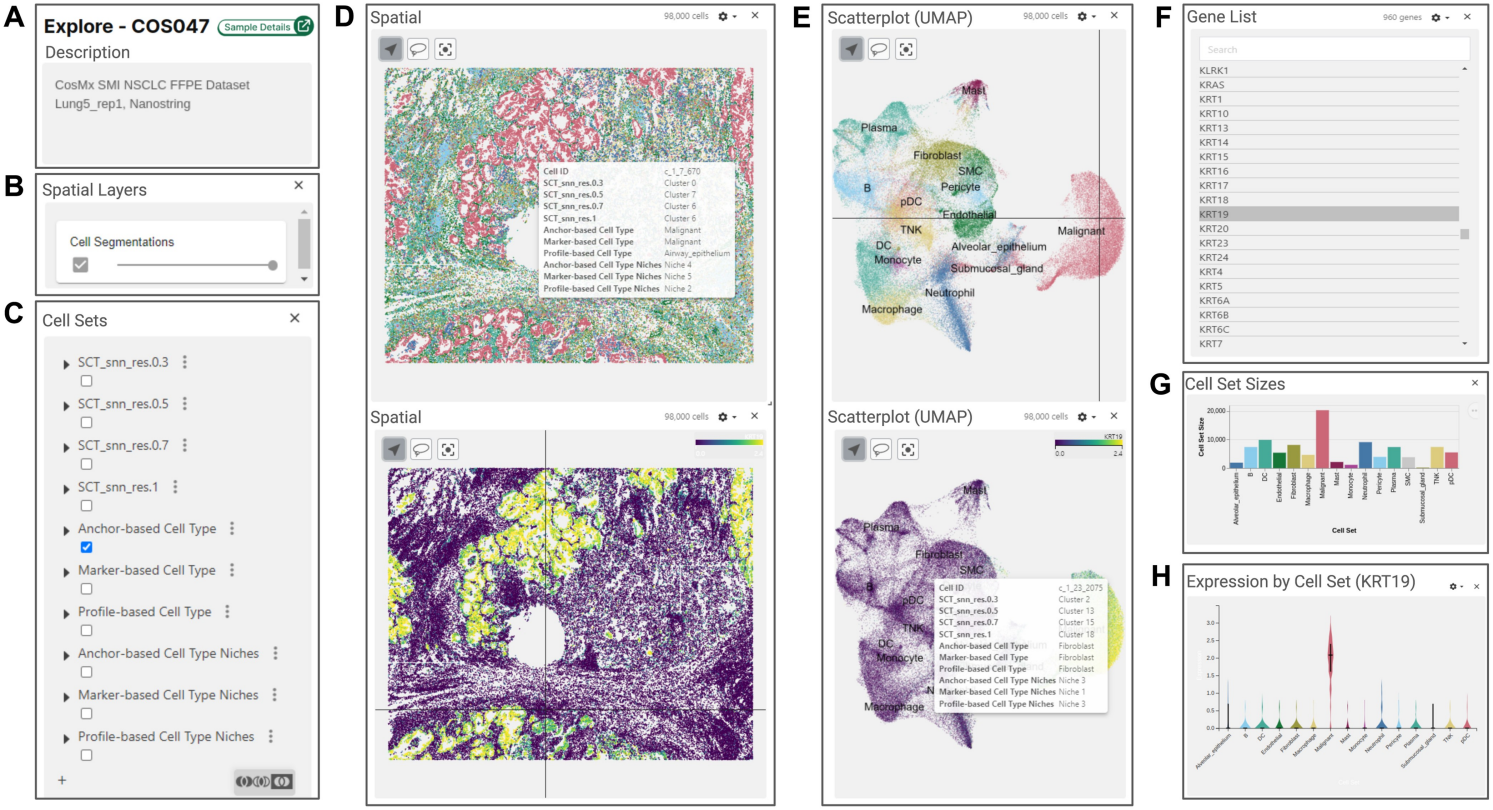
Screenshots from interactive visualization with Vitessce. Panel windows are illustrated in (A–H). (C) Cell sets panel lists 10 cell sets defined by the HISSTA analysis pipeline. Selecting one of these activates coloring the spatial and scatterplot windows by cell sets, as shown in the upper figures of (D) and (E). The lower figures show coloring by the expression values of selected gene in the gene list panel (F). The bar graph for cell set sizes shown in (G), and violin expression plots in (H) are activated by choosing cell sets or a gene of interest, respectively.

All samples and their analysis results in HISSTA can be accessed interactively through Vitessce. Information is visually presented through multiple windows, including the sample description panel, spatial layers panel, cell sets panel, spatial image panel, scatter plot panel, gene list panel, cell set sizes panel, and cell set expression panel (Fig. 5). The sample description panel shows the sample ID and name, with a link to detailed dataset information and analysis results (Fig. 5A).

The spatial layers panel includes an option for cell segmentation, allowing users to adjust the visibility of cell segmentation (Fig. 5B). The cell sets panel displays various cell sets from HISSTA analysis, including Louvain clustering results at different resolutions (0.3, 0.5, 0.7, and 1.0), cell type annotation results by three different methods, and spatial niche analysis results based on each cell type annotation method (Fig. 5C). Selecting cell sets of interest from these categories updates all accompanying windows.

HISSTA offers two main displays of cell distribution: spatial and scatterplot (UMAP) plots. The spatial panel displays a visual representation of the spatial distribution of cells (Fig. 5D), while the scatter plot panel shows the UMAP plot of cells based on gene expression values (Fig. 5E). Both windows support zooming and panning, with advanced options available in the settings icons, where cells can be colored by cell set attributes or gene expression values. The gene of interest can be selected in the gene list panel (Fig. 5F). The cell set sizes panel displays the number of cells in the selected cell sets (Fig. 5G). The expression by cell set panel shows the distribution of gene expression across selected cell sets (Fig. 5H). All these window panels are seamlessly integrated. For example, hovering over a specific cell in the spatial panel displays a tooltip detailing cell’s membership in various cell sets, while the location of the same cell in the scatterplot panel is indicated by intersecting vertical and horizontal lines (Fig. 5D and E). Interactive scanning of neighboring cells enables users to intuitively understand the association between spatial cellular composition and the tissue structure of the sample.

### 4.4 User case—a breast cancer example

To demonstrate the utility and power of interactive visualization using Vitessce, we performed an in-depth analysis for a breast cancer dataset (XEN008) produced with the 313-plex human breast Xenium panel (Janesick *et al.* 2023). The paper revealed that the sample exhibited characteristics of both mild ductal carcinoma in situ (DCIS) and invasive ductal carcinoma (IDC) through histopathological examination. It also showed that cell type composition and marker gene expression could differentiate two DCIS regions with varying levels of aggressiveness and identify molecular markers for the more aggressive regions.

**Figure 6.**
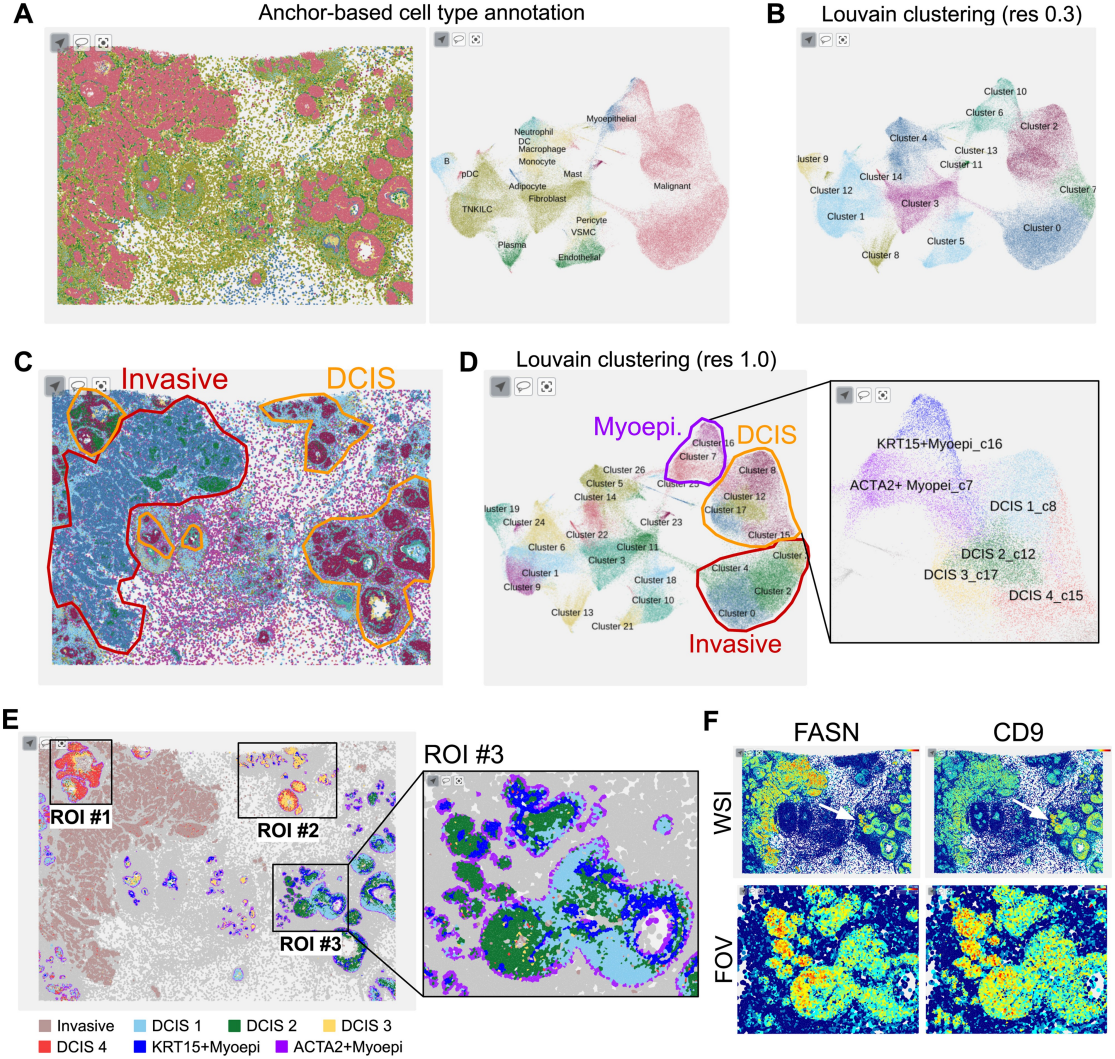
A case study for a breast cancer sample. (A) Spatial and scatterplot (UMAP) views of anchor-based cell type annotation results. (B) UMAP plot of Louvain clustering results at resolution 0.3. (C) Tissue image view with pathological assignment of disease state. Cells are colored according to the Louvain clustering results shown in (D). Area of interest is magnified for detailed assignment. (E) Regions of interest (ROIs). Cells are colored by the cell type subclusters shown in (D). (F) Tissue images colored by the expression of marker genes FASN and CD9. The upper and lower images are the whole slide image (WSI) and ROI #3, respectively.

Anchor-based cell type annotation successfully identified malignant cancer cells separate from epithelial and immune cells (Fig. 6A). Louvain clustering at resolution 0.3 further divided malignant cells into three clusters (Fig. 6B). Expression of marker genes CEACAM6, SERPINA3, AGR3, and TACSTD2 for DCIS and ABCC11, FASN, CTTN, and SQLE for IDC (Supplementary Fig. S1A and B) identified DCIS and IDC regions in the tissue image plot (Fig. 6C). To understand differences in several DCIS regions, we examined the Louvain clustering result at higher resolution 1.0, revealing four DCIS subclusters and two myoepithelial subclusters (Fig. 6D). Based on the colored tissue image, we selected three regions of interest (ROIs) (Fig. 6E). ROIs #1 and #2 were primarily mixtures of cells in subcluster DCIS3 and DCIS4, with DCIS4 cells positioned at outer rings and close to ACTA2+ myoepithelial cells. Notably, myoepithelial cells were absent in the invasive legion. ROI #3 was particularly interesting because the ductal structure was partially disrupted, with the inner parts being mixtures of cells in subclusters DCIS1 and DCIS2. The ductal boundaries are defined mainly by ACTA2+ myoepithelial cells, while KRT15+ myoepithelial cells are present inside. Furthermore, FASN, an established marker for invasive cancer cells, was highly expressed in DCIS2 cells (Fig. 6F). This suggests that DCIS2 cells may represent an intermediate state transitioning into an aggressive IDC phenotype. CD9 expression showed a strong spatial correlation with FASN expression, indicating these genes may play roles in creating tumor microenvironments that facilitate the transition to invasive cancer. Although our hypothesis certainly needs further experimental validation, supporting reports suggest that FASN-mediated changes in specific fatty acids can promote tumor cell migration (Vanauberg *et al.* 2023), and CD9 overexpression is associated with cells transitioning to a metastatic state (Li *et al.* 2023). All these analyses were performed using the HISSTA web implementation and highlight the advantages of powerful interactive visualization with Vitessce.

## 5 Conclusions and future development

The HISSTA database represents a significant advancement in the field of spatial transcriptomics by providing an extensive repository of in situ single-cell resolution data across various human tissues. The integration with interactive visualization and advanced analysis tools, such as cell type annotation, spatial colocalization, cellular communication, and niche analysis, makes HISSTA an invaluable resource for both basic research and applied studies in areas like cancer diagnosis, precision medicine, and digital pathology.

Moving forward, there are several promising directions for the future development of HISSTA. One potential area of expansion is the inclusion of single-cell proteomics data from platforms like Akoya Biosciences. By incorporating protein expression profiles at the single-cell level, HISSTA could evolve into a comprehensive multi-omics resource, providing researchers with a more holistic view of cellular processes within their spatial contexts. This would significantly enhance the utility of HISSTA, allowing for more detailed and integrative analyses of complex biological systems.

Additionally, there is a growing opportunity to leverage HISSTA in the development of more sophisticated tools for tissue analysis and diagnosis, particularly through the integration of AI-based applications. For instance, combining HISSTA's spatial transcriptomics data with machine learning models to analyze tissue images could lead to the development of advanced diagnostic tools that enhance the accuracy and efficiency of digital pathology, especially in identifying subtle patterns within tissue structures that are indicative of disease states.

Both the gene content and spatial resolution have been rapidly increasing recently. The continuous advancement of in situ imaging technologies and the growing availability of high-resolution spatial data will further drive the expansion and refinement of HISSTA. The rapid growth of relevant datasets presents another challenge in maintaining up-to-date informative resources. For example, we found that the public data available more than doubled during the last six months after the initial buildup of HISSTA. Future updates could include the incorporation of new datasets, support for additional tissues and more detailed cell type annotation, the development of more refined analytical algorithms, and the enhancement of interactive visualization tools to keep pace with the evolving needs of the research community.

In conclusion, HISSTA is not just a static database but a dynamic platform poised to play a critical role in the future of spatial biology and medicine. Its ongoing development and expansion will continue to provide researchers and clinicians with powerful tools to explore and understand the spatial complexity of biological tissues at an unprecedented level of detail.

## Supplementary Material

btaf142_Supplementary_Data

## Data Availability

The codes and data underlying this article are available at https://doi.org/10.5281/zenodo.14904523, https://kbds.re.kr/hissta/download, and https://kbds.re.kr/KAP241467/.
