# Supplementary material for: HISSTA: a human in situ single-cell transcriptome atlas
Source: Bioinformatics. 2025 Mar 31;41(4):btaf142. doi: 10.1093/bioinformatics/btaf142 (PMC12002909; doi:10.1093/bioinformatics/btaf142)
Supplement: btaf142_Supplementary_Data [file btaf142_supplementary_data.zip › HISSTA_Supplementary_data.pdf]

Supplementary data to

HISSTA: a human in situ single-cell transcriptome atlas

Jiwon Yu,[1†] Jiwoo Moon,[1†] Minseo Kim,[2†] Gyoul Han,[1] Insu Jang,[2] Jinyoung Lim,[3] Seungmook Lee,[3] Seok-Hwan Yoon,[3] Woong-Yang Park,[3, 4] Byungwook Lee,[2*] and Sanghyuk Lee[1*]

[1]Department of Life Science, Ewha Womans University, Seoul 03760, Republic of Korea

[2]Korean Bioinformation Center (KOBIC), Korean Research Institute of Bioscience and Biotechnology, Daejeon 34141, Republic of Korea

[3]Geninus Inc., 4th Fl. KDU tower, 70 Jungeui-ro, Songpa-gu, Seoul 05836, Republic of Korea

[4]GxD Inc., 6 Chome-6-2-4F Kashiwanoha, Kashiwa, Chiba 277-0882, Japan

*Corresponding authors. Department of Life Science, Ewha Womans University, Seoul 03760, Republic of Korea. E-mail: sanghyuk@ewha.ac.kr; Korean Bioinformation Center (KOBIC), Korean Research Institute of Bioscience and Biotechnology, Daejeon 34141, Republic of Korea. E-mail: bulee@kribb.re.kr.

‡Equal contribution.

--- Contents ---

Supplementary Tables

Supplementary Table S1. Compendium of scRNA-seq reference datasets

Supplementary Figures

Supplementary Figure S1. Spatial expression patterns in a breast cancer sample

Supplementary Table S1. Compendium of scRNA-seq reference datasets.

Tissue	Publication	Data source	Cohort	Donor	Status
Colon	Elmentaite et al. (2021) Nature	Cells of the human intestinal tract mapped across space and time	1	7	Normal
Lung	Sikkema et al. (2023) Nat Med	An integrated cell atlas of the lung in health and disease	11	107	Normal
Breast	Kumar et al. (2023) Nature	A spatially resolved single-cell genomic atlas of the adult human breast	3	126	Normal
Liver	Xu et al. (2023) Cell	Automatic cell-type harmonization and integration across Human Cell Atlas datasets	4	36	Normal
Kidney	Lake et al. (2023) Nature	An atlas of healthy and injured cell states and niches in the human kidney	2	45	Acute kidney failure, Chronic kidney disease, Normal
Skin	Reynolds et al. (2021) Science	Developmental cell programs ar co-opted in inflammatory skin disease	1	5	Normal
Pancreas	Xu et al. (2023) Cell	Automatic cell-type harmonization and integration across Human Cell Atlas datasets	4	20	Normal
Uterine	Marečková et al. (2023) bioRxiv	An integrated single-cell reference atlas of the human endometrium	7	64	Normal
Ovary	Vázquez-García et al. (2021) Nature	Ovarian cancer mutational processes drive site-specific immune evasion	1	33	Cancer
Lymph node	Xu et al. (2023) Cell	Automatic cell-type harmonization and integration across Human Cell Atlas datasets	4	24	Normal
Bone	Bandyopadhyay et al. (2024) Cell	Mapping the cellular biogeography of human bone marrow niches using single-cell transcriptomics and proteomic imaging	1	12	Normal
Tonsil	Massoni-Badosa et al. (2024) Immunity	An atlas of cells in the human tonsil	3	17	Normal
Stomach	Tsubosaka et al. (2023) Cell Reports	Stomach encyclopedia: Combined single-cell and spatial transcriptomics reveal cell diversity and homeostatic regulation of human stomach	1	15	Normal
Prostate	Joseph et al. (2021) The Journal of pathology	Single-cell analysis of mouse and human prostate reveals novel fibroblasts with specialized distribution and microenvironment interactions	2	9	Benign prostatic hyperplasia, Normal
Heart	Monika et al. (2020) Nature	Cells of the adult human heart	2	7	Normal
Brain	Kimberly Siletti et al. (2023) Science	Transcriptomic diversity of cell types across the adult human brain	1	4	Normal

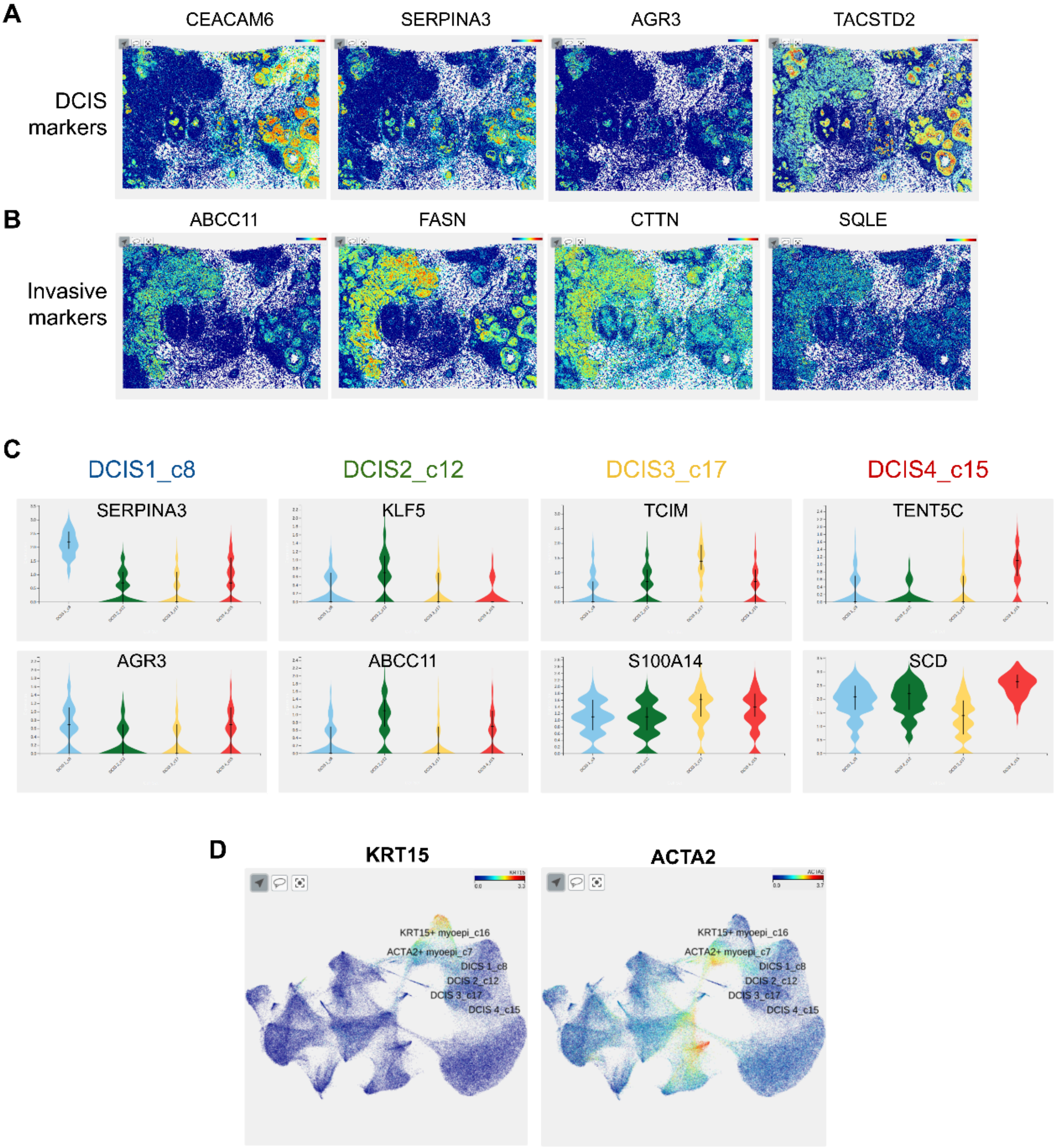

Supplementary Figure S1. Spatial expression patterns in a breast cancer sample. (**A**) Spatial distribution of DCIS markers (CEACAM6, SERPINA3, AGR3, TACSTD2) across the tissue sections. These markers highlight regions predominantly associated with DCIS. (**B**) Spatial distribution of invasive markers (ABCC11, FASN, CTTN, SQLE) indicating regions associated with invasive ductal carcinoma. (**C**) Violin plots showing the expression levels of genes with differential expression across four distinct DCIS subclusters (DCIS1_c8, DCIS2_c12, DCIS3_c17, DCIS4_c15). (**D**) UMAP visualization of myoepithelial subclusters, showing the expression patterns of KRT15 and ACTA2.
