# Supplementary figures and images for: HISSTA: a human in situ single-cell transcriptome atlas
Source: Bioinformatics. 2025 Mar 31;41(4):btaf142. doi: 10.1093/bioinformatics/btaf142 (PMC12002909; doi:10.1093/bioinformatics/btaf142)

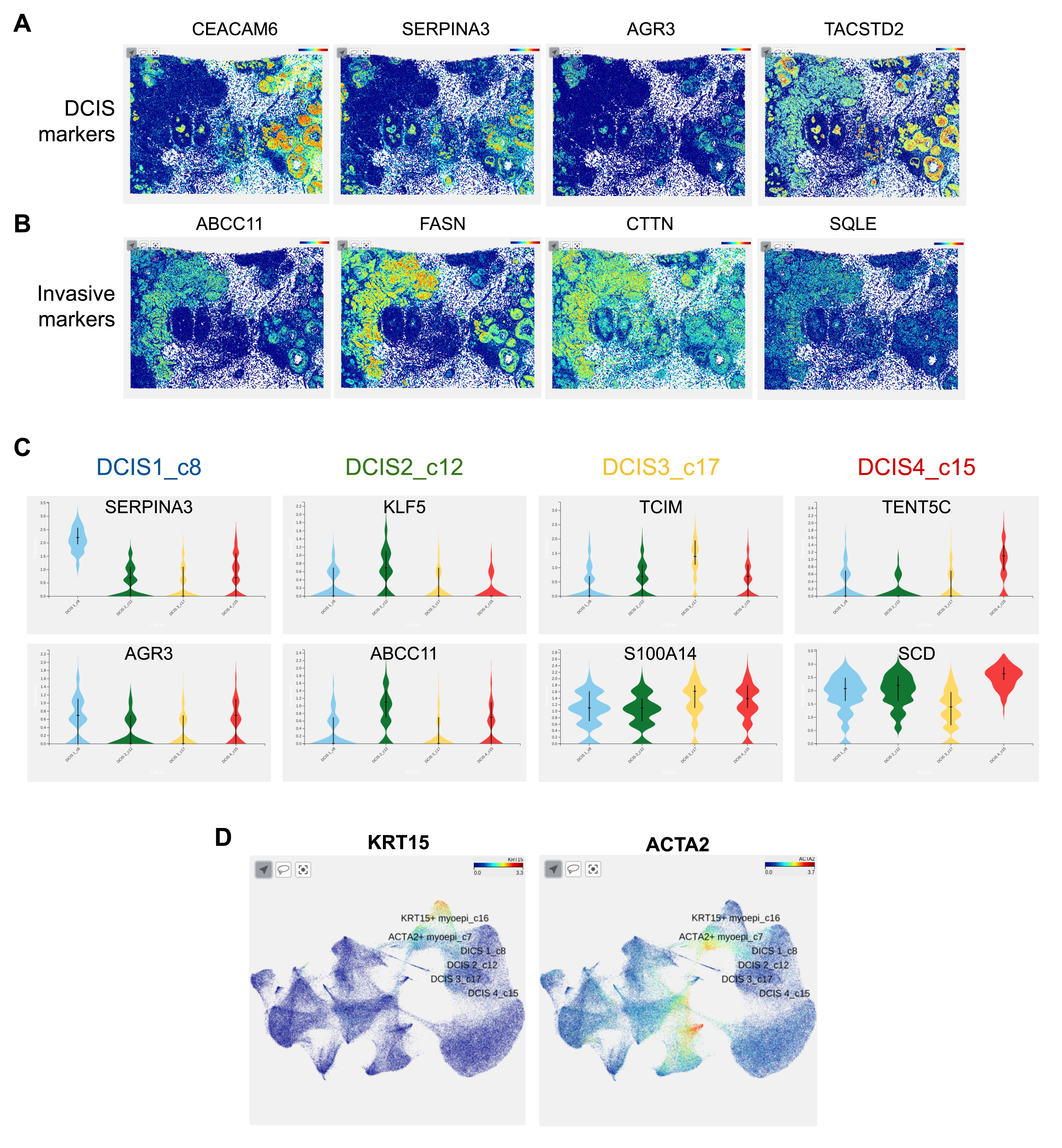

Supplement: btaf142_Supplementary_Data [file btaf142_supplementary_data.zip › supp_fig1.png]
